# Renal cancer survival and use of 5alpha-reductase inhibitors or androgen deprivation therapy

**DOI:** 10.1186/s12894-025-01923-3

**Published:** 2025-10-01

**Authors:** Olli Lipponen, Antti Pöyhönen, Andres Kotsar, Thea Veitonmäki, Teuvo LJ Tammela, Kaisa Sunela, Teemu J. Murtola

**Affiliations:** 1 Faculty of Medicine and Health Technology, Tampere University, Tampere, Finland; 2 Centre for Military Medicine, The Finnish Defence Forces, Riihimäki, Finland; 3 Department of Urology, Tartu University Hospital, Tartu, Estonia; 4 Department of Urology, TAYS Cancer Center, P.O.Box 2000, Tampere, 33521 Finland; 5 Department of Oncology, TAYS Cancer Center, Tampere, Finland

**Keywords:** Renal cancer, Survival, 5alpha-reductase inhibitors, Androgen deprivation therapy

## Abstract

**Purpose:**

Several etiological factors have been implicated in renal cell carcinoma (RCC), and hormonal receptor activity appears to influence RCC-specific mortality. This study aimed to examine the potential association between the use of 5α-reductase inhibitors and androgen deprivation therapy (ADT) and RCC-specific mortality in a population-based cohort of men.

**Methods:**

This study included a cohort of 7,720 Finnish men newly diagnosed with RCC between 1995 and 2012. The median follow-up period was 4.75 years. The risk of RCC-specific mortality associated with the use of 5α-reductase inhibitors and androgen deprivation therapy was analyzed using Cox proportional hazards regression. The influence of tumor histology and primary treatment were also evaluated. Long-term risks were assessed in lag-time analyses. Potential confounding by indication was addressed by repeating the analyses for α-blocker users for comparison.

**Results:**

Use of 5α-reductase inhibitors prior to RCC diagnosis was associated with a slight risk increase for RCC-specific mortality compared to non-users (HR 1.18, 95% CI 1.02-1.36), with similar association observed also among α-blocker users. However, post-diagnostic use of 5α-reductase inhibitors was not associated with RCC-specific mortality (HR 0.93, 95% CI 0.81-1.07). The lag time analysis did not demonstrate any long-term risk reduction for either 5α-reductase inhibitors or α-blockers. Additionally, no association was observed between ADT use and RCC-specific mortality.

**Conclusion:**

Use of 5α-reductase inhibitors or androgen deprivation therapy does not consistently associate with RCC-survival following diagnosis.

## Introduction

Renal cell cancer (RCC) consists 2.2% of all cancers globally in 2020, with 431,288 reported new cases [1]. Multiple etiological factors for RCC have been observed (e.g. smoking, obesity and hypertension, lifestyle factors, carcinogen exposure etc.) [2–4]. The overall data from literature are still inconclusive.

Before tyrosine kinase inhibitors were in use, endocrine treatments were used in the treatment of RCC, most commonly high dose progesterone. The effects of progesterone and high-dose tamoxifen were noted already in 1980s. The mechanism of action is even nowadays unclear. It has not correlated with the receptor content of the tumor tissue [5]. However, the survival rate of patients with one or more steroid receptors (estradiol, progestin or androgen) was significantly higher than patients with negative receptor status [6].

RCC is twice as common in men than in women [1, 7]. The increased morbidity in men raises a question; do androgens play a part in the pathogenesis, etiology and prognosis of RCC? In mice, castration retards the growth of androgen receptor (AR) positive human RCC tumor xenografts [8]. In this same study, it was also noted that enzalutamide therapy showed significant tumor suppression in cell lines of AR-positive RCC. The role of AR remains controversial in the pathology of RCC. It was suggested that AR could have a tumor suppressor role at least in one subtype of RCC [9]. AR expression is higher in normal renal cells compared to renal carcinoma cells. The growing amount of data supports the notion that AR expression in RCC could be oncogenic [8, 10].

5α-reductase inhibitors (5-ARIs) inhibit the reduction of testosterone into more potent dihydrotestosterone and are commonly prescribed medications used in the treatment of urinary problems and benign prostate hyperplasia. Androgen deprivation therapy (ADT) is used in the treatment of prostate cancer, usually in addition to other treatment modalities, in the treatment of advanced disease or in patients that cannot tolerate more radical treatments. Androgen deprivation can be achieved by either suppressing secretion or inhibiting the action of androgens on their receptors. Surgical and different medical treatment options exist [11].

In this retrospective population-based cohort study, we set out to examine the possible link between 5-ARI usage and RCC survival rates. We hypothesized that the patients who used 5-ARIs have a more favourable outcome regarding RCC in comparison to the patients who did not use 5-ARIs. The users of 5-ARIs are more likely under urological supervision, creating potential selection bias. To address this, we also analyzed the association of α-blockers, drug group commonly used for similar indications, with RCC survival. To further evaluate role of androgen targeted therapy as RCC prognostic factor, we evaluated the association of concurrent use of ADT to the survival of RCC.

## Materials and methods

Based on ICD-10 codes (C64), records of all renal cell cancers diagnosed in Finland beginning from 1995 to 2012 were collected from the national Finnish Cancer Registry (FCR). Accurate data on every diagnosis and histology was available, since registration of cancer diagnosis in the database is mandatory. Comprehensive data on the cause and time of death were also available [12]. Women were excluded, since 5-ARIs or ADT are not in use for women. Available data included date of RCC diagnosis, tumor extent (localized or metastatic), tumor histology, primary treatment method and the date and cause of death. Care Register for Healthcare (HILMO) data contained diagnoses and procedures recorded at hospital contacts at Finnish health care units from 1995 to 2012 [13]. The HILMO data were used to evaluate comorbidities for calculation of Charlson comorbidity index [14] and obtain information on the timing and the number of treatment methods of RCC (surgery or other oncological treatments).

The national prescription database managed by the Social Insurance Institution of Finland (SII) was linked to the study cohort to obtain data on all 5-ARI (finasteride and dutasteride) medication purchases from 1995 to 2012. Additional data on α-blocker, ADT-medications, cholesterol-lowering, antihypertensive and antidiabetic medication was also collected. Detailed information on each medication purchase, including the date of the purchase, Anatomical Therapeutic Chemical (ATC) code, package size and dose was obtained from the database.

5-ARIs, α-blockers and medications used for ADT are not available without prescription in Finland and thus they are comprehensively recorded in the database. The database does not record over-the-counter medication purchases or medications used during hospital inpatient periods. Additionally, use of finasteride 1 mg/day for androgenetic alopecia is not shown in the data.

Analyses were performed using IBM^®^ SPSS^®^ Statistics version 27. Follow-up continued from 1995 to the end of 2012 or to the death or emigration of the participant, whichever occurred first.

Cox regression was used to calculate HR and 95% CI of RCC death and death in general. Cox regression was adjusted for age at diagnosis; multivariable-adjusted model further included tumor extent, histology (clear cell vs. non-clear cell RCC), Charlson comorbidity index and primary RCC therapy (surgery vs. other). Pre-diagnostic and post-diagnostic use were analyzed in separate models. Our data on medication use started in 1995, whereas the drugs had been available earlier and our estimation on length of pre-diagnostic medication use may be inaccurate. Therefore, separate new user analysis was limited to cohort participants with no records of 5-ARI use before RCC diagnosis. In this sensitivity analysis all medication usage had started after RCC diagnosis when we had full data available. Subgroup analyses was stratified by tumor type (clear cell carcinoma vs. non-clear cell carcinomas).

Medication use was divided into two categories according to the year of purchase and the year of diagnosis of RCC, as pre-diagnostic and post-diagnostic use. Medication use on the year of RCC diagnosis and afterwards was categorized as post-diagnostic use. Pre-diagnostic use included all usage between 1995 and year of RCC diagnosis, and was analyzed as time fixed variable. Post-diagnostic use was analyzed as a time-dependent variable. According to the recorded annual drug purchases, the annual dose and the user-status updated cumulatively in separate variables for each follow-up year for every participant. Participants receiving 5-ARIs were stratified into three subgroups by calculating the additive sums of years of drugs used and the total amount of drugs used separately in the pre- and post-diagnostics periods. Tertile cut points were calculated from cumulative duration and amount of use from the entire pre- or post-diagnostic periods. Users of α-blockers were stratified similarly.

To minimize the bias associated with the clinical practice of minimizing medication use in terminal phase of cancer, post-diagnostic user status remained even after medication purchases discontinued. Both 5-ARIs and α-blockers were analyzed as a separate variable, enabling us to assess the effects of them separately as well as in concurrent use. In the ADT recipient analysis, medication use was categorized and analyzed in the same way as 5-ARI and α-blocker users.

Lag time analyses were used to evaluate latency of the risk associations and to reduce the effect of protopathic bias in cancer participants. These were performed separately for 1-, 3- and 5-year lag times respectively. In other words, in the 5-year lag time analysis, the effects of drug use 5 years earlier were estimated. E.g., exposures in 2007 were used for the endpoints occurring in 2012.

## Results

### Population characteristics

The total population size of the study was 7,720 men. The median duration of follow-up was 4.75 years after RCC diagnosis. A total number of 4,890 of the participants (63.3%) died during the follow-up. The total number of renal cancer deaths was 2,769 (35.9%).

#### ARI and alpha-blocker users before RCC diagnosis

Medication users were generally older than participants who did not use either drug. Median ages among non-drug users, 5-ARI and α-blocker recipients were 64, 75 and 73 years respectively. Crude RCC mortality was higher in 5-ARI users than α-blocker users (341/1,000 vs. 301/1,000 respectively). Compared to non-users, both 5-ARI and α-blocker had lower unadjusted all-cause and RCC mortality (Table 1).

**Table 1:** Population characteristics

	Non-user	5-ARI users		alpha-blocker users		ADT users	
		Pre-diagnosis	Post-diagnosis	Pre-diagnosis	Post-diagnosis	Pre-diagnosis	Post-diagnosis
N of participants	4740	734	1119	1238	1991	119	210
Median age (IQR) at diagnosis	64 (55–73)	75 (69–81)	71 (64–77)	73 (66–80)	69 (62–76)	69 (59–76)	67 (57–73)
Median (IQR) follow-up time	4.58 (1.00-10.08)	2.67 (0.42–5.60)	6.25 (2.83–11.17)	2.83 (0.42–5.60)	5.42 (2.50–9.83)	3.17 (0.75–5.33)	5.38 (0.98–10.33)
N (%) deaths	3244 (68.4%)	458 (62.4%)	543 (48.5%)	709 (57.3%)	1019 (51.2%)	66 (55.5%)	147 (70.0%)
Overall mortality	684/1,000	624/1,000	485/1,000	573/1,000	512/1.000	555/1,000	700/1,000
N (%) renal cell cancer deaths	1958 (41.3%)	250 (34.1%)	236 (21.1%)	373 (30.1%)	486 (24.4%)	32 (26.9%)	74 (35.2%)
Stage of cancer N (%)							
Localized	1870 (39.5%)	314 (42.8%)	640 (57.2%)	564 (45.6%)	1100 (55.2%)	53 (44.5%)	88 (41.9%)
Metastasized	1829 (38.6%)	244 (33.2%)	250 (22.3%)	396 (32.0%)	521 (26.2%)	36 (30.3%)	76 (36.2%)
Unknown	1041 (21.9%)	176 (24.0%)	229 (20.5%)	278 (22.4%)	370 (18.6%)	30 (25.2%)	46 (21.9%)
Primary treatment N (%)							
Curative Surgery	1991 (42.0%)	318 (43.3%)	678 (60.6%)	559 (45.2%)	1190 (59.8%)	64 (53.8%)	98 (46.7%)
Other	2749 (58.0%)	416 (56.7%)	441 (39.4%)	679 (54.8%)	801 (40.2%)	55 (46.2%)	112 (53.3%)

#### ARI and alpha-blocker users after RCC diagnosis

Medication users were generally younger than in the pre-diagnostic group. Median age for the 5-ARI users were 71 years and α-blocker users were two years younger. 5-ARI and α-blocker recipients had a lower all-cause mortality (485/1,000 and 512/1,000) than participants who did not use either drug. Same was true in the RCC specific mortality (211/1,000 and 244/1,000). Non-users had higher all-cause and RCC specific mortality (684/1,000 and 413/1,000 respectively) (Table 1).

#### ADT users

Total of 274 men were present in the cohort with a median age of 68 years (IQR 57–74). RCC specific mortality was 269/1,000 in pre-diagnostic and 352/1,000 in post-diagnostic setting.

### Risk of renal cancer death by use of 5-ARIS and α-blockers

#### Renal cancer death by pre-diagnostic medication use

Compared to non-users, users of 5-ARIs showed a slightly increased risk of renal cancer death on multivariable adjusted analysis (HR 1.18, 95% CI 1.02–1.36). Significant increase in risk was observed with the total amount of years of drug used and the total amount of drug used (Over 4 years of drug used, HR 1.35, 95% CI 1.05–1.74 and over 980 total doses, HR 1.29, 95% CI 1.02–1.63 respectively) (Table 2).

**Table 2:** Risk of renal cancer death by use of 5-ARIs and alpha blockers before and after diagnosis

Pre-diagnostic use						
	5-ARI users			alpha-blocker users		
Medication use	N of participants/deaths	HR (95% CI) age adjusted	HR (95% CI) multivariable adjusted^a^	N of participants/deaths	HR (95% CI) age adjusted	HR (95% CI) multivariable adjusted*
Non-user		Ref	Ref		Ref	Ref
Any use	734/250	1.05 (0.91–1.22)	1.18 (1.02–1.36)	1238/373	0.99 (0.88–1.11)	1.12 (0.99–1.26)
Duration of use:		Median 2 years			Median 2 years	
1. tertile	251/90	1.03 (0.83–1.28)	1.15 (0.92–1.43)	526/164	0.96 (0.81–1.13)	1.10 (0.93–1.30)
2. tertile	283/94	1.00 (0.81–1.25)	1.11 (0.89–1.38)	388/121	1.02 (0.84–1.24)	1.19 (0.98–1.45)
3. tertile	200/66	1.15 (0.89–1.48)	1.35 (1.05–1.74)	324/88	1.02 (0.81–1.28)	1.07 (0.85–1.34)
Amount of use:		Median 478 doses			Median 210 doses	
1. tertile	253/92	1.06 (0.85-1-31)	1.24 (0.99–1.54)	436/119	0.82 (0.68–0.99)	0.96 (0.80–1.17)
2. tertile	240/81	0.99 (0.79–1.25)	1.01 (0.80–1.27)	401/139	1.15 (0.96–1.38)	1.26 (1.05–1.51)
3. tertile	241/77	1.06 (0.84–1.34)	1.29 (1.02–1.63)	401/115	1.06 (0.86–1.29)	1.18 (0.96–1.44)
Post-diagnostic use						
	5-ARI users			alpha-blocker users		
Medication use	N of participants/deaths	HR (95% CI) age adjusted	HR (95% CI) multivariable adjusted^a^	N of participants/deaths	HR (95% CI) age adjusted	HR (95% CI) multivariable adjusted*
Non-user		Ref	Ref		Ref	Ref
Any use	1119/236	0.77 (0.67–0.88)	0.93 (0.81–1.07)	1991/486	0.65 (0.57–0.74)	0.76 (0.66–0.87)
Duration of use:		Median 2 years			Median 2 years	
1. tertile	448/131	0.82 (0.68–0.98)	0.90 (0.75–1.08)	896/306	0.86 (0.76–0.97)	0.99 (0.87–1.12)
2. tertile	340/69	0.85 (0.66–1.08)	0.96 (0.75–1.22)	616/130	0.68 (0.56–0.81)	0.87 (0.73–1.05)
3. tertile	331/36	0.60 (0.43–0.83)	0.93 (0.66–1.31)	479/50	0.43 (0.32–0.57)	0.55 (0.41–0.74)
Amount of use:		Median 400 doses			Median 270 doses	
1. tertile	375/111	0.99 (0.82–1.20)	1.07 (0.88–1.30)	702/225	0.85 (0.74–0.98)	0.99 (0.86–1.14)
2. tertile	371/84	0.72 (0.57–0.89)	0.80 (0.64-1.00)	652/189	0.82 (0.70–0.95)	1.01 (0.87–1.18)
3. tertile	373/41	0.58 (0.42–0.79)	0.88 (0.64–1.22)	637/72	0.42 (0.33–0.53)	0.54 (0.42–0.69)

^a^Calculated using Cox regression with model adjustment for age at diagnosis, tumor stage at diagnosis (localized vs. metastatic), primary RCC treatment (surgery vs. other), RCC histology (clear cell vs. non-clear cell) and Charlson comorbidity index

Similar risk association was observed in α-blocker users on multivariable adjusted analysis (HR 1.12, 95% CI 0.99–1.26) (Table 2). No association was observed regarding the years of drug used or the amount of drug used.

#### Renal cancer death by post-diagnostic medication use

In the age adjusted analysis, a significant risk reduction for RCC specific death was observed among 5-ARI users compared t non-users (HR 0.77, 95% CI 0.67–0.88) with an inverse association with cumulative 5-ARI dose (Table 2). However, the association did not remain after on multivariable adjustment (HR 0.93, 95% CI 0.81–1.07).

In α-blocker users a significant risk reduction was observed in multivariable analysis (HR 0.76, 95% CI 0.66–0.87). Age adjusted analysis showed a greater risk reduction in RCC specific death (HR 0.65, 95% CI 0.57–0.74) that correlated inversely with the increasing dose. Dependency on the duration of use was also observed with a similar inverse correlation. This trend was not evident in 5-ARI users (Table 2).

#### Renal cancer death by ADT use

When compared to non-users, those who used ADT before RCC diagnosis (*n* = 119, 1.5% of total population) showed no significant risk association with RCC death in multivariable adjusted analysis (HR 1.12, 95% CI 0.79–1.59). In post-diagnostic setting (*n* = 210, 2.7%), non-significantly increased HR for RCC death was observed (HR 1.20, 95% CI 0.84–1.72) (Table 3).

**Table 3:** Risk of renal cancer death by the use of ADT

Pre-diagnostic use			
	ADT users		
Medication use	N of participants/deaths	HR (95% CI) age adjusted	HR (95% CI) multivariable adjusted^a^
Non-user		Ref	Ref
Any use	119/32	0.98 (0.69–1.40)	1.12 (0.79–1.59)
Post-diagnostic use			
	ADT users		
Medication use	N of participants/deaths	HR (95% CI) age adjusted	HR (95% CI) multivariable adjusted^a^
Non-user		Ref	Ref
Any use	210/74	1.35 (0.94–1.93)	1.20 (0.84–1.72)

^a^Calculated using Cox regression with model adjustment for age at diagnosis, tumor stage at diagnosis (localized vs. metastatic), primary RCC treatment (surgery vs. other), RCC histology (clear cell vs. non-clear cell) and Charlson comorbidity index

### Lag time analysis

Compared to non-users, 5-ARI users showed a minor reduction in the risk of RCC death with 1-year time-lag (HR 0.85,95% CI 0.72-1.00). This remained unchanged with 3-year and 5-year lag time, However, risk estimate did not differ compared to α-blocker users. No significant risk difference by ADT use was observed in lag time analyses.

### Sensitivity analyses

In the new user analysis 5-ARI usage after diagnosis was associated with a a reduction in RCC specific mortality on the multivariable adjusted analysis (HR 0.72, 95% CI 0.59–0.88) and the age adjusted analysis (HR 0.56, 95% CI 0.46–0.68). (Table 4) The α-blocker users had similar risk association, with no clear difference between the two drug groups (Table 4).

**Table 4:** Risk of renal cancer death by new use of 5-ARIs or alpha-blockers after RCC diagnosis

Post-diagnostic use				
	5-ARI users		alpha-blocker users	
Medication use	HR (95% CI) age adjusted	HR (95% CI) multivariable adjusted^a^	HR (95% CI) age adjusted	HR (95% CI) multivariable adjusted^a^
Non-user	Ref	Ref	Ref	Ref
Any use	0.56 (0.46–0.68)	0.72 (0.59–0.88)	0.63 (0.54–0.74)	0.76 (0.65–0.88)
Duration of use				
1. tertile	0.54 (0.41–0.73)	0.64 (0.48–0.85)	0.86 (0.75–0.98)	1.00 (0.87–1.15)
2. tertile	0.71 (0.51–0.99)	0.81 (0.58–1.13)	0.68 (0.56–0.84)	0.90 (0.74–1.11)
3. tertile	0.50 (0.33–0.76)	0.78 (0.51–1.20)	0.42 (0.31–0.58)	0.55 (0.40–0.75)
Amount of use				
1. tertile	0.65 (0.49–0.85)	0.74 (0.56–0.98)	0.84 (0.72–0.97)	1.00 (0.86–1.17)
2. tertile	0.51 (0.35–0.75)	0.60 (0.41–0.89)	0.83 (0.70–0.98)	1.02 (0.86–1.21)
3. tertile	0.54 (0.34–0.79)	0.83 (0.56–1.22)	0.43 (0.33–0.56)	0.55 (0.42–0.72)

^a^Calculated using Cox regression with model adjustment for age at diagnosis, tumor stage at diagnosis (localized vs. metastatic), primary RCC treatment (surgery vs. other), RCC histology (clear cell vs. non-clear cell) and Charlson comorbidity index

### Subgroup analyses

#### Primary treatment

Primary treatment modality did not modify the risk association between 5-ARI users and RCC death neither for the pre- nor post-diagnostic usage. In multivariable adjusted analysis HR for RCC death by post-diagnostic 5-ARI use managed with radical curative-intent surgery as a primary therapy 0.91, 95% CI 0.71–1.16. For the RCC patients who underwent other therapies as primary treatment, no risk reduction was observed by 5-ARI user, either (HR 0.92, 95% CI 0.78–1.10) (Table 5).

**Table 5:** Risk of renal cancer death in different primary treatment modalities by use of 5-ARIs and alpha-blockers

Surgery					Other primary treatment				
Pre-diagnostic use					Pre-diagnostic use				
	5-ARI users		alpha-blocker users			5-ARI users		alpha-blocker users	
Medication use	HR (95% CI) age adjusted	HR (95% CI) multivariable adjusted^a^	HR (95% CI) age adjusted	HR (95% CI) multivariable adjusted^a^	Medication use	HR (95% CI) age adjusted	HR (95% CI) multivariable adjusted^a^	HR (95% CI) age adjusted	HR (95% CI) multivariable adjusted^a^
Non-user	Ref	Ref	Ref	Ref	Non-user	Ref	Ref	Ref	Ref
Any use	1.09 (0.80–1.49)	1.18 (0.86–1.61)	0.87 (0.67–1.14)	0.90 (0.69–1.18)	Any use	1.11 (0.94–1.30)	1.20 (1.02–1.41)	1.09 (0.95–1.25)	1.15 (1.01–1.32)
Duration of use					Duration of use				
1. tertile	1.08 (0.69–1.70)	1.16 (0.73–1.84)	0.99 (0.72–1.36)	0.98 (0.71–1.37)	1. tertile	1.14 (0.89–1.46)	1.20 (0.94–1.54)	1.06 (0.87–1.28)	1.12 (0.92–1.35)
2. tertile	1.19 (0.76–1.85)	1.31 (0.84–2.06)	0.71 (0.43–1.15)	0.82 (0.50–1.34)	2. tertile	1.04 (0.81–1.34)	1.10 (0.85–1.41)	1.08 (0.88–1.33)	1.18 (0.96–1.46)
3. tertile	1.06 (0.55–2.01)	1.07 (0.57–2.04)	0.77 (0.44–1.35)	0.73 (0.42–1.28)	3. tertile	1.13 (0.85–1.49)	1.35 (1.02–1.78)	1.16 (0.91–1.49)	1.19 (0.92–1.52)
Amount of use					Amount of use				
1. tertile	1.26 (0.83–1.90)	1.38 (0.90–2.11)	0.93 (0.65–1.32)	0.95 (0.66–1.36)	1. tertile	1.14 (0.89–1.47)	1.26 (0.98–1.63)	0.86 (0.68–1.07)	0.92 (0.74–1.16)
2. tertile	1.09 (0.66–1.83)	1.11 (0.66–1.87)	0.87 (0.57–1.34)	0.92 (0.60–1.41)	2. tertile	1.02 (0.78–1.32)	1.04 (0.80–1.34)	1.26 (1.03–1.54)	1.29 (1.06–1.57)
3. tertile	0.90 (0.50–1.63)	1.02 (0.57–1.83)	0.78 (0.47–1.28)	0.78 (0.47–1.30)	3. tertile	1.12 (0.87–1.45)	1.29 (1.00-1.67)	1.20 (0.96–1.49)	1.28 (1.03–1.60)
Post-diagnostic use					Post-diagnostic use				
	5-ARI users		alpha-blocker users			5-ARI users		alpha-blocker users	
Medication use	HR (95% CI) age adjusted	HR (95% CI) multivariable adjusted^a^	HR (95% CI) age adjusted	HR (95% CI) multivariable adjusted^a^	Medication use	HR (95% CI) age adjusted	HR (95% CI) multivariable adjusted^a^	HR (95% CI) age adjusted	HR (95% CI) multivariable adjusted^a^
Non-user	Ref	Ref	Ref	Ref	Non-user	Ref	Ref	Ref	Ref
Any use	0.83 (0.65–1.06)	0.91 (0.71–1.16)	0.79 (0.61–1.02)	0.82 (0.63–1.05)	Any use	0.82 (0.69–0.97)	0.92 (0.78–1.10)	0.73 (0.62–0.85)	0.78 (0.66–0.91)
Duration of use					Duration of use				
1. tertile	0.65 (0.42–0.99)	0.66 (0.43–1.02)	0.98 (0.76–1.25)	1.03 (0.80–1.32)	1. tertile	0.91 (0.74–1.11)	0.98 (0.80–1.20)	0.97 (0.85–1.12)	1.02 (0.89–1.18)
2. tertile	1.12 (0.79–1.59)	1.16 (0.82–1.65)	0.80 (0.60–1.06)	0.82 (0.62–1.09)	2. tertile	0.80 (0.57–1.13)	0.79 (0.56–1.11)	0.79 (0.62-1.00)	0.91 (0.71–1.16)
3. tertile	0.78 (0.51–1.21)	0.95 (0.62–1.48)	0.72 (0.51–1.01)	0.76 (0.53–1.07)	3. tertile	0.57 (0.33–0.99)	0.85 (0.49–1.48)	0.24 (0.14–0.42)	0.29 (0.17–0.52)
Amount of use					Amount of use				
1. tertile	0.91 (0.62–1.34)	0.89 (0.60–1.30)	0.98 (0.76–1.26)	1.03 (0.80–1.33)	1. tertile	1.09 (0.87–1.37)	1.17 (0.94–1.47)	1.00 (0.85–1.19)	1.04 (0.88–1.23)
2. tertile	0.87 (0.58–1.30)	0.94 (0.63–1.40)	0.92 (0.69–1.23)	0.94 (0.70–1.26)	2. tertile	0.72 (0.55–0.94)	0.74 (0.57–0.97)	0.93 (0.77–1.11)	1.04 (0.87–1.25)
3. tertile	0.77 (0.51–1.17)	0.94 (0.62–1.42)	0.64 (0.47–0.88)	0.68 (0.49–0.93)	3. tertile	0.52 (0.31–0.86)	0.71 (0.43–1.18)	0.33 (0.22–0.48)	0.39 (0.26–0.58)

^a^Calculated using Cox regression with model adjustment for age at diagnosis, tumor stage at diagnosis (localized vs. metastatic), primary RCC treatment (surgery vs. other), RCC histology (clear cell vs. non-clear cell) and Charlson comorbidity index

#### Tumor histology

When analysis was stratified by RCC histology (Clear cell RCC vs. non-clear cell RCC), no significant effect modification could be observed for the risk of RCC death among 5-ARI users. (Table 6).

**Table 6:** Risk of RCC death in clear cell renal cell carcinoma (ccRCC) versus non-ccRCC by use of 5-ARIs and alpha-blockers

ccRCC					Non-ccRCC				
Pre-diagnostic use					Pre-diagnostic use				
	5-ARI users		alpha-blocker users			5-ARI users		alpha-blocker users	
Medication use	HR (95% CI) age adjusted	HR (95% CI) multivariable adjusted^a^	HR (95% CI) age adjusted	HR (95% CI) multivariable adjusted^a^	Medication use	HR (95% CI) age adjusted	HR (95% CI) multivariable adjusted^a^	HR (95% CI) age adjusted	HR (95% CI) multivariable adjusted^a^
Non-user	Ref	Ref	Ref	Ref	Non-user	Ref	Ref	Ref	Ref
Any use	1.08 (0.91–1.27)	1.19 (1.01–1.40)	0.99 (0.86–1.15)	1.16 (1.00-1.33)	Any use	1.12 (0.71–1.77)	1.45 (0.89–2.35)	0.80 (0.53–1.23)	1.07 (0.69–1.67)
Duration of use					Duration of use				
1. tertile	0.98 (0.76–1.27)	1.09 (0.84–1.40)	0.94 (0.78–1.14)	1.12 (0.92–1.36)	1. tertile	1.60 (0.84–3.03)	2.20 (1.16–4.15)	0.79 (0.44–1.42)	1.24 (0.69–2.22)
2. tertile	1.10 (0.86–1.41)	1.23 (0.96–1.57)	1.08 (0.86–1.34)	1.31 (1.05–1.63)	2. tertile	0.73 (0.35–1.53)	0.73 (0.33–1.62)	1.01 (0.55–1.84)	1.47 (0.78–2.74)
3. tertile	1.15 (0.86–1.54)	1.30 (0.97–1.73)	0.98 (0.75–1.28)	1.05 (0.81–1.36)	3. tertile	1.65 (0.71–3.80)	3.98 (1.67–9.49)	0.63 (0.26–1.50)	0.85 (0.34–2.10)
Amount of use					Amount of use				
1. tertile	0.99 (0.76–1.27)	1.16 (0.90–1.51)	0.81 (0.65–1.02)	1.01 (0.80–1.26)	1. tertile	1.62 (0.85–3.10)	2.37 (1.20–4.69)	0.66 (0.33–1.30)	0.88 (0.45–1.75)
2. tertile	1.10 (0.84–1.42)	1.09 (0.84–1.41)	1.13 (0.92–1.40)	1.29 (1.05–1.59)	2. tertile	0.82 (0.37–1.83)	0.95 (0.43–2.14)	1.16 (0.63–2.13)	1.79 (0.95–3.37)
3. tertile	1.12 (0.85–1.46)	1.32 (1.02–1.73)	1.09 (0.86–1.37)	1.20 (0.95–1.51)	3. tertile	0.92 (0.42-2.00)	1.47 (0.66–3.29)	0.70 (0.34–1.43)	0.80 (0.38–1.71)
Post-diagnostic use					Post-diagnostic use				
	5-ARI users		alpha-blocker users			5-ARI users		alpha-blocker users	
Medication use	HR (95% CI) age adjusted	HR (95% CI) multivariable adjusted^a^	HR (95% CI) age adjusted	HR (95% CI) multivariable adjusted^a^	Medication use	HR (95% CI) age adjusted	HR (95% CI) multivariable adjusted^a^	HR (95% CI) age adjusted	HR (95% CI) multivariable adjusted^a^
Non-user	Ref	Ref	Ref	Ref	Non-user	Ref	Ref	Ref	Ref
Any use	0.78 (0.66–0.91)	0.94 (0.81–1.11)	0.65 (0.55–0.75)	0.75 (0.64–0.88)	Any use	0.64 (0.37–1.10)	0.91 (0.52–1.59)	0.73 (0.46–1.15)	1.00 (0.63–1.61)
Duration of use					Duration of use				
1. tertile	0.81 (0.66-1.00)	0.91 (0.74–1.12)	0.87 (0.76-1.00)	0.99 (0.86–1.14)	1. tertile	0.55 (0.25–1.18)	0.65 (0.30–1.41)	0.65 (0.41–1.02)	0.95 (0.60–1.51)
2. tertile	0.88 (0.67–1.16)	0.96 (0.73–1.27)	0.70 (0.57–0.87)	0.90 (0.73–1.11)	2. tertile	0.94 (0.38–2.35)	1.53 (0.59–3.97)	0.67 (0.34–1.35)	0.94 (0.45–1.93)
3. tertile	0.60 (0.41–0.88)	0.92 (0.62–1.35)	0.43 (0.31–0.61)	0.59 (0.42–0.83)	3. tertile	0.77 (0.24–2.45)	1.54 (0.47–5.05)	0.37 (0.13–1.01)	0.63 (0.22–1.76)
Amount of use					Amount of use				
1. tertile	0.98 (0.79–1.22)	1.11 (0.89–1.38)	0.84 (0.71–0.98)	0.96 (0.81–1.12)	1. tertile	0.73 (0.32–1.66)	0.74 (0.32–1.70)	0.64 (0.36–1.12)	0.92 (0.52–1.66)
2. tertile	0.73 (0.57–0.94)	0.79 (0.61–1.02)	0.87 (0.73–1.04)	1.05 (0.89–1.25)	2. tertile	0.67 (0.29–1.54)	1.13 (0.48–2.68)	0.80 (0.48–1.33)	1.12 (0.66–1.90)
3. tertile	0.56 (0.38–0.80)	0.83 (0.57–1.20)	0.44 (0.33–0.58)	0.58 (0.44–0.77)	3. tertile	0.59 (0.19–1.87)	1.15 (0.35–3.73)	0.31 (0.13–0.76)	0.49 (0.19–1.22)

^a^Calculated using Cox regression with model adjustment for age at diagnosis, tumor stage at diagnosis (localized vs. metastatic), primary RCC treatment (surgery vs. other), RCC histology (clear cell vs. non-clear cell) and Charlson comorbidity index

## Discussion

We did not observe reduction in the risk of renal cancer death by 5-ARI use. Both 5-ARI users and α-blocker users showed similar trends in HRs and no clear difference could be observed between the drug groups. This suggests that the observed risk differences could be caused indirectly by the underlying differences between men with and without symptomatic BPH which prompted the medication use, rather than directly by use of any specific drug group. The observed HRs were not more favourable for 5-ARI users. This does not support prognostic role of 5-ARIs in RCC.

The new user analysis showed a slightly clearer risk reduction in 5-ARI users in comparison to non-users. However, the risk reduction was similar also in the α-blocker users and thus speaks against the effects of 5-ARIs behind it. Significant results were observed in the lag time analysis, but again the CIs of 5-ARIs and α-blockers were superimposed. It is likely that selection is behind these findings because of the similar risk association profiles for both 5-ARI and α-blocker users is evident. The results of our sensitivity analyses support these findings, even though a slightly more favourable risk estimates were evident in the new-user analysis.

No correlation could be established with ADT and the prognosis of RCC in this study. The sample size was relatively small (*n* = 274, 3.5%), thus our ability to study the risk of RCC death by ADT use was limited. It has been demonstrated that intratumoral steroidogenesis is a significant source of androgens in AR-positive RCC [8]. ADT works by inhibiting androgen production in the testes, but probably not in the renaltumorals. It could be speculated, that androgen receptor pathway inhibitors (ARPIs) like enzalutamide and abiraterone could affect intratumoral androgen production. However, they were not in clinical use at the time of our study period, and should be assessed in further studies.

To our knowledge, this is the first study to examine the association between the use of 5-ARI, ADT and survival in RCC. Our findings are consistent with previous studies, although epidemiological studies on this topic remain limited [15, 16]. Tissue, cell line and functional studies have suggested that androgen signaling axis is pro-tumorigenic in RCC [8, 17]. Therefore, medications that suppress this signaling pathway could have a favourable clinical effect.

The role of AR remains controversial in RCC. Positive AR expression in male RCC patients has been linked with lower pathological grade and earlier tumor stage [17]. It also associates with improved survival especially in clear cell RCC [9]. On the other hand, AR has also been suggested to promote RCC tumorigenesis via dysregulation of non-coding RNAs [18]. In vitro, AR activation with DHT promotes RCC proliferation [19]. Due to this controversy, further research is required to further clarify roles of AR in RCC and possible therapeutic efficacy of AR targeted drugs.

Our study shows that drugs affecting systemic androgen level or DHT production locally in the prostate do not associate with RCC survival. Our study was not adequately powered to investigate survival outcomes for specific RCC subtypes, and thus potential benefits in certain subgroups may have gone undetected. Future studies are needed to elucidate whether inhibition of local androgen production in RCC cells with ARPIs would lead to survival benefits.

Lead time bias could also explain our findings, as the indication for 5-ARI medication is treatment of lower urinary tract symptoms (LUTS) and benign prostate hyperplasia. Men with LUTS have frequent urological check-ups, which might lead to an earlier RCC diagnosis at a more curable stage, generating lead time bias. This is supported by the fact that the users of α-blockers showed similar risk reduction profiles as the 5-ARI users, even though the pharmacological mechanism of action is different.

Strength of our study include the comprehensive and accurate data on drug purchases and RCC deaths available from the national databases. The comprehensive information on the duration and amount of drug use enabled the separate analysis of pre- and post-diagnostic medication, as well as the analysis of time and dose dependencies of RCC death.

As our study is a retrospective study without randomization, confounding due to background factors might have a marked effect on the results. This is evident in age difference, drug users were generally older than non-users. Alpha-blocker medication users were younger than 5-ARI users. As younger patients can be expected to have better prognosis this can create bias. Other factors include lifestyle factors, smoking, physical activity and socioeconomic status of the participants. As these factors were unknown to us, we could not evaluate the possible confounding effects these might have had.

To our knowledge, this is the first study to assess the effect of 5-ARI or ADT administration on the prognosis of RCC. Men receiving these medications did not have a better disease-specific survival after RCC diagnosis than non-users. Our study does not support the role of systemic androgen suppression or local inhibition in the prostate in the progression of RCC.

## Data Availability

“The study protocol was reviewed and approved by the ethics committee of the Pirkanmaa hospital district, as well as by the administrators of each health care registry used in this study. The study utilized solely routinely collected data from national health-care registries, approvals and consents were obtained from registry keepers, Finnish Institute for Health and Welfare (THL) and Social Insurance Institution of Finland (SII).In compliance with Finnish law on medical research (9.4.1999/488) IRB approval can be waived for studies based entirely on routinely collected registry data and no approval or waiver of ethics approval are needed, nor granted. The datasets generated and analyzed during the current study are not publicly available due it includes personal level data and specific permission from national authority FINDATA is needed to access the data by Finnish law, but are available from the corresponding author on reasonable request.”

## Abbreviations

ADT: Androgen deprivation therapy
AR: Androgen receptor
ARPI: Androgen receptor pathway inhibitor
CI: Confidence interval
DHT: Dihydrotestosterone
HR: Hazard ratio
LUTS: Lower urinary tract symptoms
RCC: Renal cell cancer
5-ARIs: 5α-reductase inhibitors
